# Malignancies in inborn errors of immunity: a retrospective-prospective study on a large Italian cohort of pediatric and adult patients

**DOI:** 10.3389/fimmu.2026.1948296

**Published:** 2026-09-24

**Authors:** Cinzia Milito, Carmen D’Anzieri, Francesco Cinetto, Chiara Pagnozzi, Maria Carrabba, Giovanna Fabio, Maria Giovanna Danieli, Martina Bartolucci, Giuseppe Spadaro, Gianluca Lagnese, Caterina Cancrini, Beatrice Rivalta, Andrea Finocchi, Carolina Marasco, Angelo Vacca, Francesca Conti, Mattia Moratti, Viviana Moschese, Mayla Sgrulletti, Antonino Trizzino, Silvia Ricci, Lorenzo Lodi, Alessandro Aiuti, Federica Barzaghi, Claudio Pignata, Emilia Cirillo, Marco Gattorno, Serena Palmeri, Luisa Brussino, Stefania Nicola, Davide Firinu, Giulia Costanzo, Eleonora Gambineri, Alberto Tommasini, Samuele Naviglio, Simone Cesaro, Matteo Chinello, Irene D’Alba, Lucia Augusta Baselli, Giorgio Costagliola, Francesca Fioredda, Francesco Gigliotti, Adele Civino, Sonia Bonamoni, Alfonso Piciocchi, Gigliola Di Matteo, Baldassarre Martire

**Affiliations:** 1 Department of Molecular Medicine, “Sapienza” University of Rome, Rome, Italy; 2 Department of Translational and Precision Medicine, “Sapienza” University of Rome, Rome, Italy; 3 Rare Diseases Referral Center, Internal Medicine 1, Ca’ Foncello Hospital, AULSS2 Marca Trevigiana, Treviso, Italy; 4 Department of Medicine - DIMED, University of Padova, Padova, Italy; 5 Department of Medical Area, Fondazione IRCCS Ca’ Granda Ospedale Maggiore Policlinico, ERN-RITA Center, Milan, Italy; 6 SOS Immunologia delle Malattie Rare e dei Trapianti, Dipartimento di Medicina Interna, Azienda Ospedaliero Universitaria delle Marche, and Università Politecnica delle Marche, Ancona, Italy; 7 Postgraduate School of Allergy and Clinical Immunology, Università Politecnica delle Marche, Ancona, Italy; 8 Department of Translational Medical Sciences, University of Naples Federico II, Naples, Italy; 9 Research and Clinical Unit of Primary Immunodeficiencies, IRCCS Bambino Gesù Children’s Hospital, Rome, Italy; 10 Department of System Medicine, Pediatric Chair, University of Tor Vergata, Rome, Italy; 11 Clinical Unit of Immuno-Infettivology IRCCS Bambino Gesù Children’s Hospital, Rome, Italy; 12 Unit of Internal Medicine “Guido Baccelli”, Department of Precision and Regenerative Medicine and Ionian Area (DiMePRe-J), University of Bari, Aldo Moro Medical School, Bari, Italy; 13 Pediatric Unit, IRCCS Azienda Ospedaliero-Universitaria di Bologna, Department of Medical and Surgical Sciences, Alma Mater Studiorum, University of Bologna, Bologna, Italy; 14 Pediatric Unit, IRCCS Azienda Ospedaliero-Universitaria di Bologna, Bologna, Italy; 15 Department of Systems Medicine, University of Tor Vergata, Rome, Italy; 16 Pediatric Immunopathology and Allergology Unit, Policlinico Tor Vergata, University of Rome Tor Vergata, Rome, Italy; 17 Department of Pediatric Hematology and Oncology, ARNAS Ospedali Civico Di Cristina Benfratelli Hospital, Palermo, Italy; 18 Immunology Unit, Department of Pediatrics, Meyer Children’s Hospital IRCCS, Florence, Italy; 19 San Raffaele Telethon Institute for Gene Therapy, IRCCS San Raffaele Scientific Institute, Milan, Italy; 20 Vita-Salute San Raffaele University, Milan, Italy; 21 Department of Translational Medical Sciences - Section of Pediatrics, University of Naples Federico II, Naples, Italy; 22 Department of Neurosciences, Rehabilitation, Ophthalmology, Genetics, Maternal and Child Health (DINOGMI), University of Genoa, Genoa, Italy; 23 Rheumatology and Autoinflammatory Unit, IRCCS G. Gaslini, Genoa, Italy; 24 Department of Medical Sciences, University of Torino, Torino, Italy; 25 Allergy and Clinical Immunology Unit, Mauriziano Hospital, Torino, Italy; 26 Department of Medical Sciences and Public Health, University of Cagliari, Cagliari, Italy; 27 Department of NEUROFARBA, Section of Child Health, University of Florence, Meyer Children’s Hospital, IRCCS, Florence, Italy; 28 Institute for Maternal and Child Health IRCCS “Burlo Garofolo”, Trieste, Italy; 29 Pediatric Hematology Oncology, Department of Mother and Child, Azienda Ospedaliera Universitaria Integrata, Verona, Italy; 30 Paediatric Haematology-Oncology, Maternal Infant Hospital ‘G. Salesi’, Ancona, Italy; 31 Pediatric Immunorheumatology Unit, Fondazione IRCCS Ca’ Granda Ospedale Maggiore Policlinico, Milan, Italy; 32 Section of Pediatric Hematology and Oncology, Azienda Ospedaliero-Universitaria Pisana, Pisa, Italy; 33 Unit of Hematology, IRCCS G. Gaslini, Genoa, Italy; 34 Renato Dulbecco University Hospital, Catanzaro, Italy; 35 Pediatric Rheumatology and Immunology Unit, ‘Vito Fazzi’ Hospital, Lecce, Italy; 36 Department of Pediatrics, Fondazione IRCCS San Gerardo, Monza, Italy; 37 GIMEMA Foundation, Rome, Italy; 38 Department of Systems Medicine, University of Rome Tor Vergata, Rome, Italy; 39 Pediatrics and Neonatology Unit, Maternal-Infant Department, “Monsignor A.R. Di Miccoli” Hospital, Barletta, Italy

**Keywords:** CVID, hematologic cancers, inborn errors of immunity, malignancy, solid cancers

## Abstract

**Background:**

Inborn Errors of Immunity (IEI) are associated with an increased risk of malignancy due to impaired immune surveillance, genetic instability and chronic inflammation, However, comprehensive epidemiological data from large national cohorts remain limited.

**Methods:**

We conducted a multicenter, retrospective-prospective observational study involving 4,540 pediatric and adult patients with IEI, followed across 30 Italian referral centers, with the aim of evaluating those who developed cancer. Clinical, immunological, genetic, and oncological data were prospectively collected from October 2023 to October 2025, while the retrospective period covered January 2000 to September 2023. These findings were then compared with data from Italian population-based cancer registries (AIRTUM/AIRC).

**Results:**

Out of 4,540 patients with IEI, 248 developed malignancies: 133 females and 115 males, comprising 195 adults and 53 children. The overall prevalence of malignancy within the IEI cohort was 5.5% (95% CI: 4.8–6.2), with an incidence rate of 3.4 cases per 1,000 person-years. Common variable immunodeficiency represented the largest IEI subgroup and showed the highest prevalence of malignancy 11.7%, (95% CI: 10.1–13.4). Malignancies occurred significantly earlier than in the general Italian population, at a median age of 44 years (range: 1–82), particularly in patients with ataxia-telangiectasia, 8.5 years (range 4-23). In contrast, patients with IgA deficiency displayed the highest median age at first malignancy, 52 years (range: 18- 57), compared to other IEI categories. In 30.2% of cases, cancer represented the first clinical manifestation leading to the IEI diagnosis. A different oncological profile compared to Italian population-based data was observed, with Non-Hodgkin lymphoma (21.7%) as the most frequent cancer, followed by skin cancers (11.2%) and gastric adenocarcinoma (10.4%), The overall 10-year survival rate was 94%, with no significant differences between hematologic and solid malignancies, or between adult and pediatric cohorts.

**Conclusions:**

The multicenter, registry-based design of our study enabled a reliable estimation of cancer incidence among IEI patients and a direct comparison with the general population, while the longitudinal dataset provided valuable insights into the cancer spectrum and outcomes across different IEI subtypes. Tailored surveillance strategies, early IEI diagnosis and multidisciplinary management are essential to improve long-term outcomes.

## 1 Introduction

Inborn Errors of Immunity (IEI) encompass a broad and heterogeneous group of disorders arising from defects in the development, maturation or function of the immune system. Currently, these conditions are associated with mutations in 508 different genes and 17 known phenocopies (1, 2). Besides an increased susceptibility to infections and immune dysregulation disorders, IEIs exhibit a significant predisposition to malignancy. Following infections, cancer is the second leading cause of mortality in both pediatric and adult patients and may represent the initial clinical manifestation in a substantial number of cases (3, 4).

Recent studies have demonstrated that the same molecular defects underlying IEI can also directly promote oncogenesis (5–7). Key pathogenic mechanisms include impaired immunosurveillance, defective DNA repair, chronic inflammation, and infection by oncogenic viruses such as Epstein–Barr virus (EBV), human herpesvirus (HHV), and human papillomavirus (HPV) (8–11). Furthermore, patients with immune regulatory disorders - such as Autoimmune Lymphoproliferative Syndrome (ALPS), activated PI3Kδ syndrome (APDS type 1), and IL-10 receptor deficiency - are particularly predisposed to lymphomas, reflecting specific defects in apoptosis and immune checkpoints (12, 13).

Data from the ESID registry indicates that the prevalence of malignancies in the IEI cohort is 1.78 times higher than in the general European population (8.9% vs. 5%) (14, 15). Malignancy occurs in approximately 8–10% of IEI patients, conferring an overall cancer risk 1.42 to 2.3-fold higher than that of the general population (15). Lymphomas account for nearly two-thirds of all cases, particularly non-Hodgkin B-cell lymphoma (B-NHL), followed by leukemia and epithelial tumors (16). Among IEI subtypes, Common Variable Immunodeficiency (CVID) and combined immunodeficiencies associated with DNA repair defects (e.g., Ataxia-telangiectasia) exhibit the highest prevalence, whereas complement deficiencies and Chronic Granulomatous Disease (CGD) appear to confer minimal to no increased oncological risk (17, 18). In addition, oncological outcomes in IEI patients are generally worse than in immunocompetent subjects, due to increased treatment-related toxicities, reduced efficacy of standard therapeutic regimens, and/or higher risks of relapse and secondary malignancies (12, 16). Based on these considerations, we conducted a multicenter retro-prospective observational study to evaluate adult and pediatric patients with IEI in the Italian cohort who had been diagnosed with cancer.

## 2 Materials and methods

### 2.1 Study design and patient population

The study involved a large national cohort of 4,540 patients with Inborn Errors of Immunity (IEI). The study population included both adult and pediatric subjects (≤ 18 years) followed at 30 Italian Referral Centers within Italian Network for Primary Immunodeficiency (IPINET). Data were prospectively collected from October 2023 to October 2025, while retrospective data covered the period from January 1, 2000, to September 30, 2023.

The diagnosis of IEI for all enrolled patients was established based on the European Society for Immunodeficiencies (ESID) diagnostic criteria (19) and categorized according to the 2024 International Union of Immunological Societies (IUIS) classification (1, 2). The study cohort specifically comprised IEI patients who developed a neoplastic disease either prior to or following their IEI diagnosis.

### 2.2 Data collection and variables

Comprehensive clinical data were collected for each participant, including: demographic characteristics (sex, date of birth); age at IEI diagnosis; and molecular diagnosis, where available. We recorded IEI-associated comorbidities, such as allergies, autoimmunity, enteropathy, pulmonary involvement and growth failure. Details regarding IEI-specific therapies were also documented, including immunoglobulin replacement, biological agents, immunosuppressive drugs, cytokine growth factors and anti-infective prophylaxis.

Regarding oncological features, data included: age at cancer diagnosis, tumor histological subtype, family history of malignancy and potential oncological risk factors. Furthermore, we recorded infections present at the time of cancer diagnosis and the therapeutic interventions received (surgery, chemotherapy, radiotherapy, immunotherapy and hematopoietic stem cell transplantation). Clinical outcomes were assessed based on the date of the last follow-up, vital status (alive/deceased) and, where available, the date and cause of death.

### 2.3 Ethical considerations

The study was approved by the local Ethics Committee of the Azienda Ospedaliero Universitaria “Policlinico Foggia” (CE Interprovinciale Area 1, Prot. 8/Seg.CE/24) and conducted in accordance with the Good Clinical Practice (GCP) guidelines, the International Conference on Harmonization (ICH) standard and the latest revision of the Declaration of Helsinki. Written informed consent was obtained from all adult patients and from the legal guardians of pediatric participants enrolled in the prospective phase.

### 2.4 Statistical analysis

Demographic and clinical characteristics were summarized using descriptive statistics. Continuous variables are expressed as median and range, whereas categorical variables are reported as absolute frequencies and percentages. Comparisons between adult and pediatric cohorts, as well as between different IEI diagnostic categories, were performed using the Mann–Whitney U test for continuous variables and the Chi-square or Fisher’s exact test for categorical variables, as appropriate. All statistical tests were two-sided, with a p-value < 0.05 considered statistically significant. Analyses were performed using R software (version 4.3).

The prevalence of malignancy within the IEI cohort was calculated overall and for major diagnostic subgroups, such as Common Variable Immunodeficiency (CVID). These values were compared with expected cancer incidence rates and cancer-type distribution in the general Italian population using normative data from the AIRTUM (Italian Association of Cancer Registers) and AIRC (Italian Association for Cancer Research) databases. AIRTUM is a coordinated network of population-based cancer registries that collects incidence and survival data across 20 geographic areas in Italy. We utilized the 2024 AIRTUM and AIRC reports to estimate the expected oncological burden in the age-matched general population. Results are presented as crude rates and percentages to highlight deviations from population-based estimates (20, 21). Survival (SUR) probability was calculated, by the Kaplan–Meier method, comparisons between probabilities in different patient groups were performed using the log-rank test. Results were expressed as probability and 95% confidence interval (95% CI).

## 3 Results

### 3.1 Cohort characteristics

During the study period, 248 out of 4,540 enrolled patients (5.5%) developed one malignancy, as detailed in **Figure 1a**. This subgroup comprised 195 (79%) adults and 53 (21%) pediatric patients. The adult cohort included 114 (58.5%) females and 81 (41.5%) males, while the pediatric cohort showed a male predominance with 34 (64%) males and 19 (36%) females. The median age at IEI diagnosis was 39 years (range: 0–75) for the entire cohort, 43 years (range: 19–75) and 8 years (range: 0–18) for the adult and pediatric groups, respectively. The mean duration of follow-up per patient with a tumor was 16 years (**Table 1****).**

**Figure 1:**
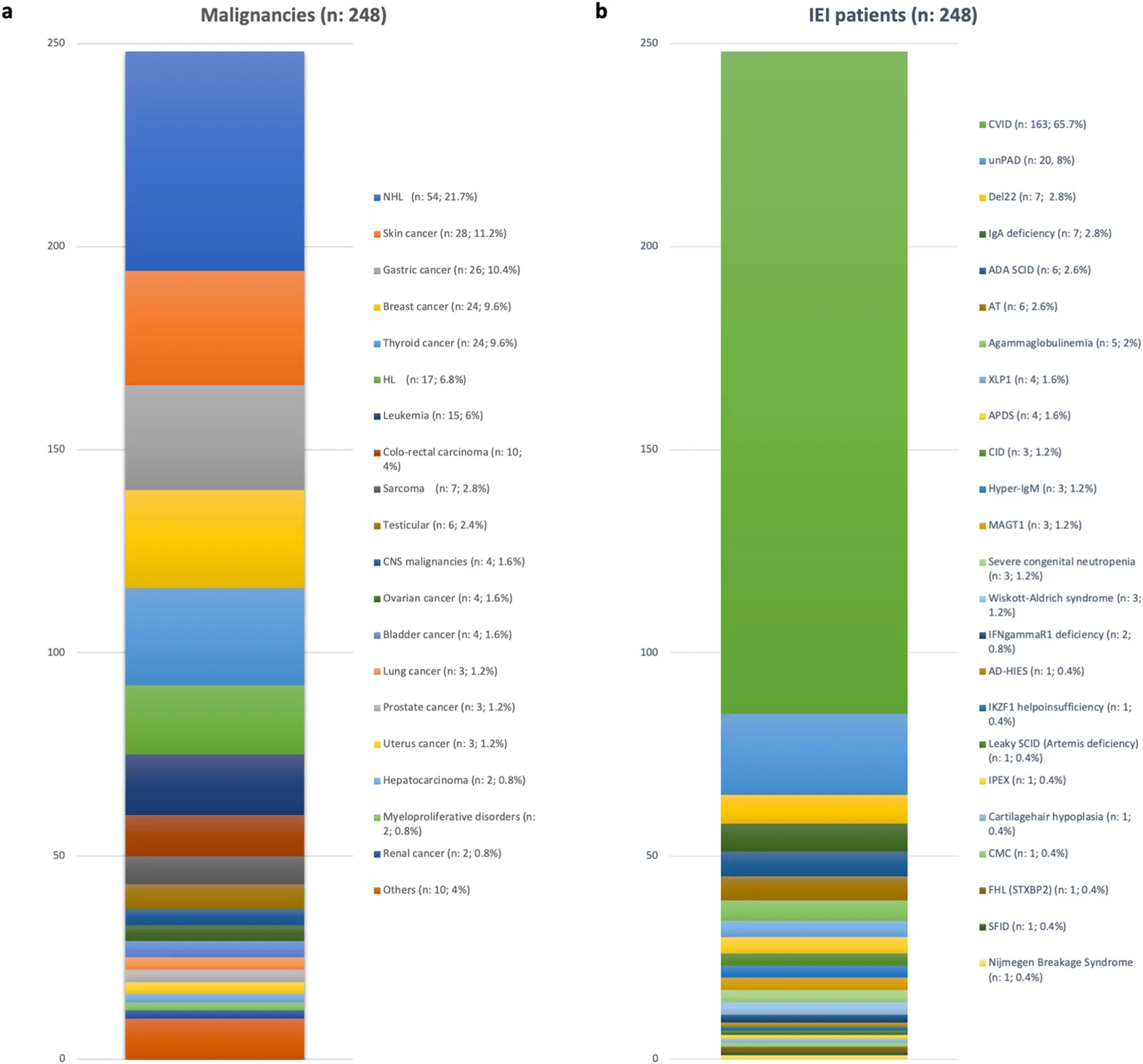
Distribution of malignancies **(a)** and IEIs **(b)** in the whole cohort of 248 patients. APDS, Activated phosphoinositide 3-kinase delta Syndrome; AT, ataxia telangiectasia; CID, combined immunodeficiency; CMC, Chronic Mucocutaneous Candidiasis; CNS, central nervous system; CVID, common variable immunodeficiency; Del22, 22q11.2 deletion syndrome; FHL, Familial HLH; HIES, Hyper-IgE syndrome; HL, Hodgkin’s lymphoma; IPEX, Immunedysregulation, Polyendocrinopathy, Enteropathy, X-linked; NHL, non-Hodgkin’s lymphoma; SCID, severe combined immunodeficiency; SFID, Sideroblastic anemia with immunodeficiency, fevers and developmental delay; unPAD, unclassified primary antibody deficiency; XLP-1, X-linked lymphoproliferative disease type 1.

**Table 1:** Demographic and clinical features of the entire cohort of patients developing malignancies and of the adult and pediatric sub-cohorts.

Features	Overall patients N = 248	Adult N = 195	Pediatric N = 53	p-value
Sex, n (%)	F 133 (54%)	F 115 (59%)	F 19 (36%)	0.005
	M 115 (46%)	M 80 (41%)	M 34 (64%)	<0.001
Median age (y) at diagnosis of IEI, (min, max)	39 (0-75)	43 (19-75)	8 (0-18)	<0.001
N° of cancer, n (%)	248	195 (78.6%)	53 (21.3%)	<0.001
Median age at diagnosis of cancer, median (min, max)	44 (1.82)	49 (19-82)	10 (1-18)	<0.001
Patients with IEI-associated comorbidities, n (%)	170 (68.5%)	137 (70%)	33 (62%)	0.26
Cancers’ familiarity, n (%)	65 (26%)	60 (30%)	5 (9.4%)	0.0017
Infections at the time of diagnosis of the tumor, n (%)	41 (16.5%)	27 (13.8%)	14 (26.4%)	0.029
Outcomes at the end of study	Alive 227 (94.5%)	185 (94.9%)	42 (79.2%)	0.14
	Dead 13 (5.4%)	8 (4.1%)	5 (9.4%)	
	Unknown 8 (3.3%)	2 (1%)	6 (11.3%)	
Median age (y) at death, (min-max)	38 (7-76)	59 (29-76)	12 (7-26)	<0.001

Comparisons between adult and pediatric cohorts, as well as between different IEI diagnostic categories, were performed using the Mann–Whitney U test for continuous variables and the Chi-square or Fisher’s exact test for categorical variables, as appropriate. All statistical tests were two-sided, with a p-value < 0.05 considered statistically significant.

### 3.2 IEI categories of patients developing malignancies

In the overall cohort, the most frequent underlying IEI was Common Variable Immunodeficiency (CVID), accounting for 65.7% of cases (n163), followed by Unclassified Antibody Deficiency (UnPAD) at 8% (n = 20). Both 22q11.2 deletion syndrome and selective IgA deficiency accounted for 2.8% (7 patients each). The detailed distribution of IEI subtypes is illustrated in **Figure 1b**.

### 3.3 Malignancy burden and timing

A total of 248 malignancies were diagnosed, with each patient developing a distinct tumor, comprising 195 cases in adults and 53 in pediatric patients. The overall prevalence of malignancy within the IEI cohort was 5.5% (95% CI: 4.8–6.2%), with an incidence rate of 3.4 cases per 1,000 person-years, with CVID and UnPAD being the most common categories among cancer patients, as expected due to their numerical predominance within the study cohort. The median age at the time of cancer diagnosis for the entire cohort was 42 years (range: 1–78) (**Figure 2a**)- specifically 49 years (range: 19–78) for adults and 10 years (range: 1–18) for pediatric patients, respectively. As expected, the age at cancer diagnosis varied significantly according to the IEI subtype: patients with IgA deficiency had a higher median age, 52 years (range 18-57) whereas those with syndromic features or DNA repair defects were diagnosed significantly earlier, with median ages of 11years (range 1-33) for 22q11.2 deletion syndrome (Del 22) and 8.5 years (range 4-23) for Ataxia-Telangiectasia (AT) (**Figure 2b**). The median interval between IEI diagnosis and the onset of malignancy was 10 years (range: 0–55) for the whole cohort, specifically 11 years (range: 1–55) in adults and 5 years (range: 0–17) in children. In 75 patients (30.2%), 56 adults and 19 children, malignancy was the initial clinical manifestation that led to the IEI diagnosis. Following the onset of cancer, an underlying IEI was suspected based on family history, the presence of immune dysregulation diseases, or associated viral infections, particularly by EBV. Within this subgroup, the most frequent underlying IEIs were Common Variable Immunodeficiency (CVID, 59%), Unclassified PAD (UnPAD, 14.6%), and selective IgA deficiency (5.3%).

**Figure 2:**
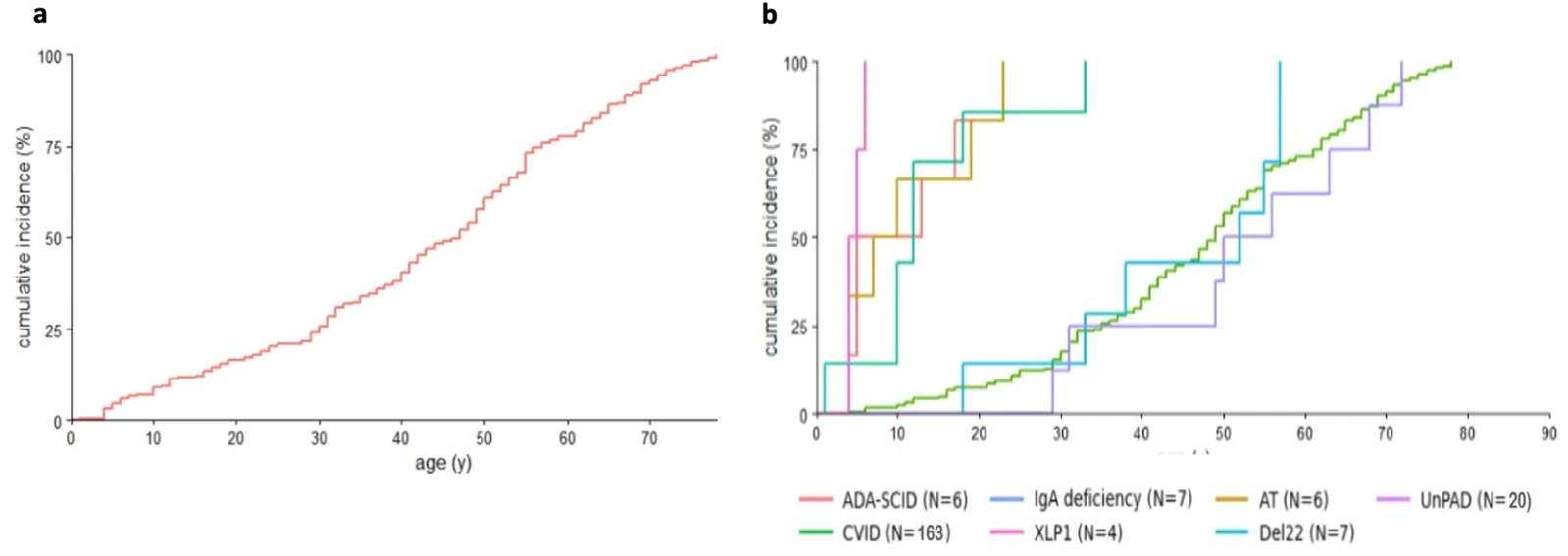
Age at malignancy in the whole cohort **(a)** and per IEI category **(b)**. The median age at the time of the cancer diagnosis for the entire cohort was 42 years (range: 1–78)-**(a)**. Patients with IgA deficiency had a higher median age, 52 years (range 18-57). Individuals with syndromic features or DNA repair defects were diagnosed significantly earlier, with median ages of 11years (range 1-33) for 22q11.2 deletion syndrome (Del 22) and 8.5 years (range 4-23) for Ataxia-Telangiectasia (AT) respectively-**(b)**. AT, ataxia telangiectasia; CVID, common variable immunodeficiency; Del22, 22q11.2 deletion syndrome; SCID, severe combined immunodeficiency; unPAD, unclassified primary antibody deficiency; XLP-1, X-linked lymphoproliferative disease type 1.

### 3.4 Spectrum of malignancies

Within the entire cohort, NHL represented the most prevalent malignancy, accounting for 54 cases (21.7%). This was followed by skin cancers—including both melanoma and non-melanoma types—with 28 cases (11.2%), gastric adenocarcinoma with 26 cases (10.4%), and breast and thyroid carcinomas, each presenting in 24 patients (9.6%). Additionally, the cohort included 17 cases of Hodgkin lymphoma (HL; 6.8%) and 15 cases of acute leukemia (6%). The latter comprised 12 lymphoblastic and 3 myeloid leukemias, the vast majority of which occurred within the pediatric subgroup (13/15, 86.7%). When comparing the pediatric and adult cohorts, a marked difference in tumor distribution was observed (**Figure 3**). Hematologic malignancies predominated in the pediatric cohort, accounting for 68% of cases, whereas solid tumors were most common among adult patients, representing 70% of all diagnoses. The comprehensive distribution of malignancies according to IEI diagnosis is illustrated in **Figure 4**. Notably, in the entire cohort, the median age at diagnosis of malignancy was 37 years (range: 4.0–82.0) for NHL and 47 (range: 19.0–77.9) for gastric cancer, values that are substantially lower than the approximately 70 years observed in the general Italian population for both tumors.

**Figure 3:**
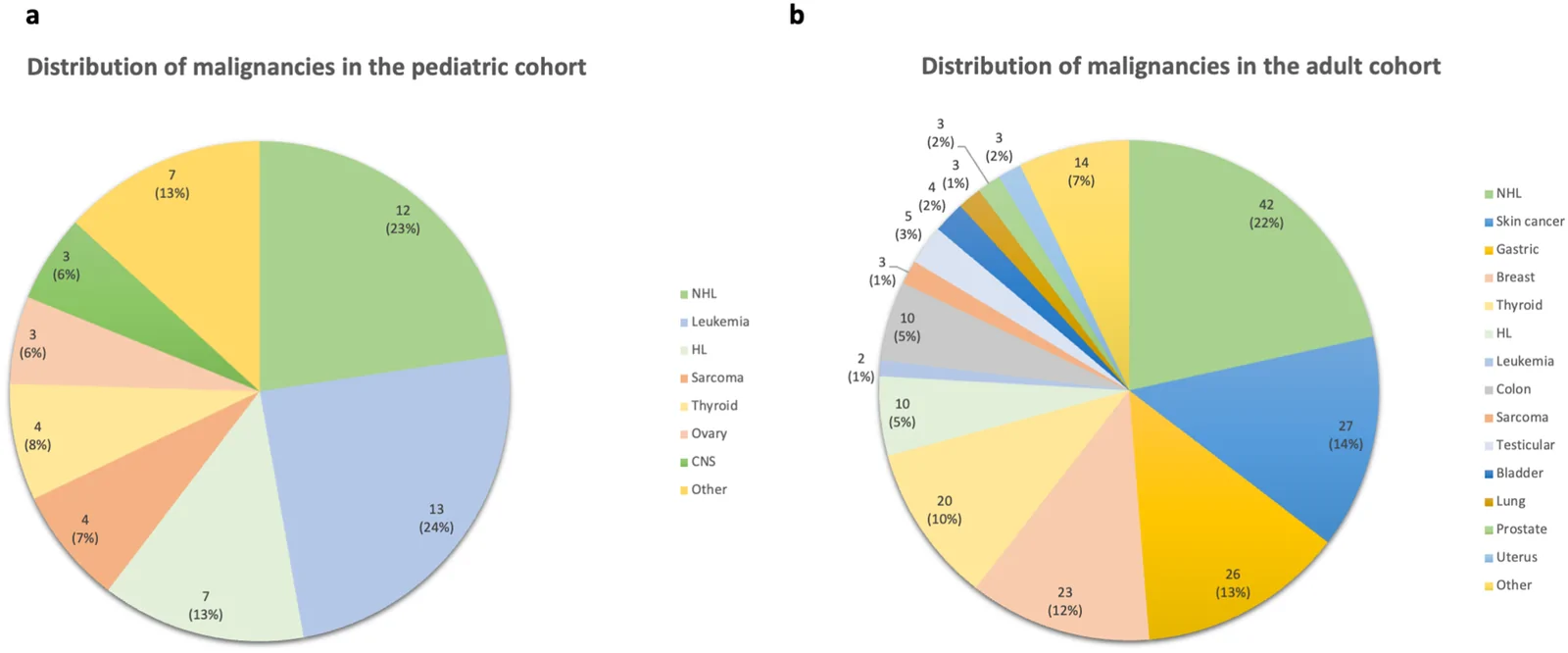
Distribution of malignancies in the pediatric **(a)** and adult cohort **(b)**. HL, Hodgkin’s lymphoma; NHL, non-Hodgkin’s lymphoma; CNS, Central Nervous System.

**Figure 4:**
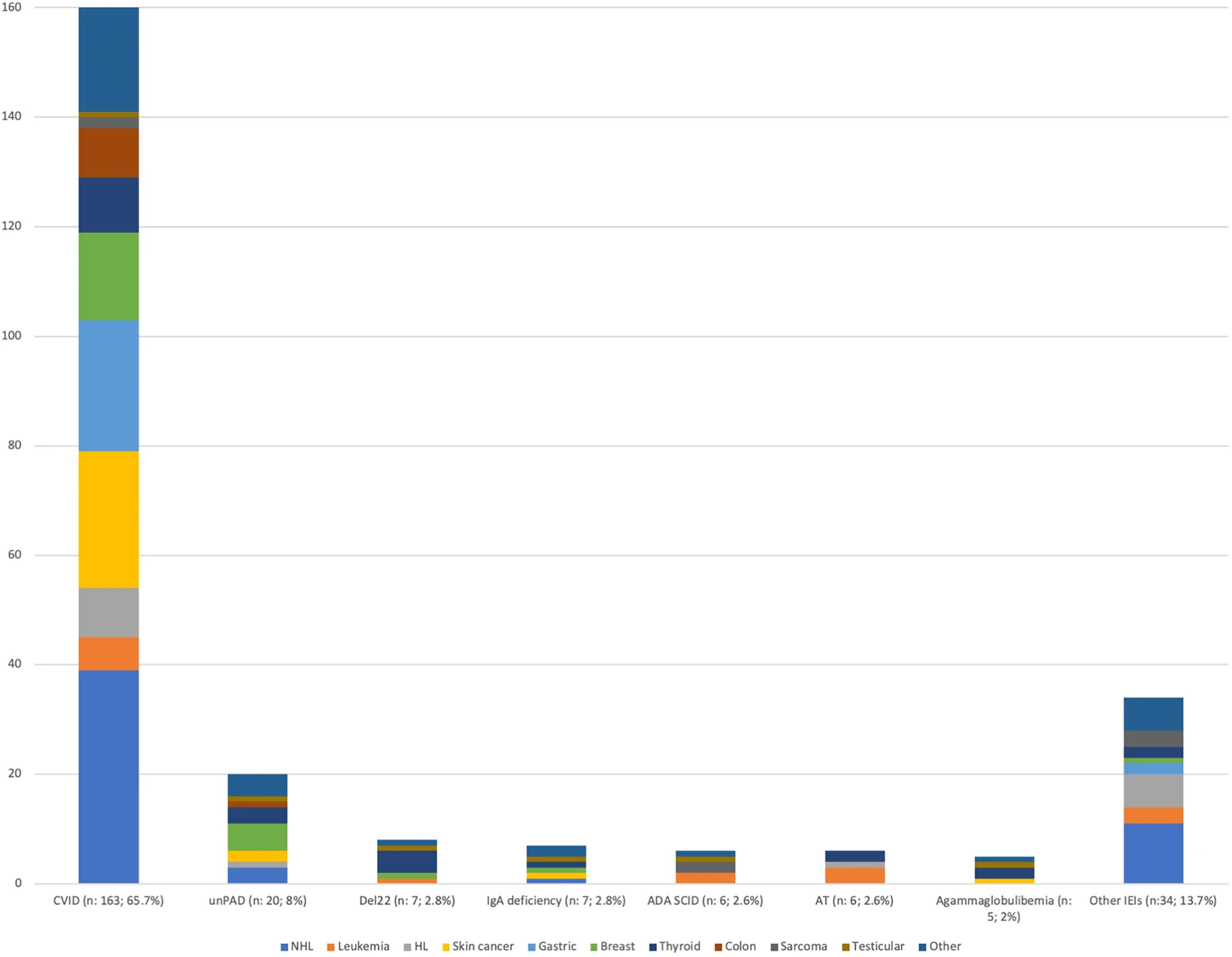
Cancer distribution by IEI subtype. AT, ataxia telangiectasia; CVID, common variable immunodeficiency; Del22, 22q11.2 deletion syndrome; HL, Hodgkin’s lymphoma; NHL, non-Hodgkin’s lymphoma; SCID, severe combined immunodeficiency; unPAD, unclassified primary antibody deficiency.

### 3.5 Risk factors, concurrent infections and comorbidities

A positive family history of cancer was recorded in 65/248 patients developing malignancies, with a higher frequency in adults compared to pediatric patients (60 vs. 5; p< 0.05). Identifiable risk factors were present in 66 patients, with smoking reported by 30% of the overall cohort.

At the time of cancer diagnosis, 16.5% of patients were found to have concurrent infections. At the time of cancer diagnosis, 16.5% of patients presented with concurrent infections. The majority of these infections were viral, including EBV, CMV, HCV, and HHV-8, though bacterial infections, specifically Helicobacter pylori, were also observed.

A statistically significant difference was observed between the adult and pediatric cohorts (13.8% vs. 26.4%; p< 0.05), particularly with regard to viral infections which were more prevalent in children (24.5% vs. 9.7%; p< 0.05)(**Table 1**).

Associated IEI-related comorbidities were present in 170/248 patients developing malignancies (68.5%; 137 adults and 33 children). Since multiple patients presented with multi-organ involvement and concurrent manifestations, the cumulative prevalence of each manifestation was calculated relative to the overall cohort. These included pulmonary diseases (87, 35%), autoimmune hematologic disorders—either isolated or combined cytopenias—and autoimmune non-hematologic disorders (77 patients, 31% each), allergic diseases (23, 9.3%), and growth failure (5.2%). Among the organ-specific autoimmune manifestations, the most frequently involved organs were the gastrointestinal tract (26%), thyroid (17%), and skin (12%). Regarding pulmonary comorbidities, granulomatous-lymphocytic interstitial lung disease (GLILD) and bronchiectasis were the most frequent (26% and 24%, respectively).

### 3.6 Genetic and cytogenetic findings

Genetic and cytogenetic findings were collected from 69 of the 248 patients with diagnosis of malignancy (comprising 30 adults and 39 pediatric patients). Pathogenic or likely pathogenic variants were identified in 52 patients. The most frequently identified mutated genes were ATM and ADA among all patients affected by these IEIs (6 each) (**Table 2**). Cancer-related germline or somatic cytogenetic alterations were detected in 17 patients (6 adults and 11 pediatric patients). Among the germline cytogenetic alterations, the 22q11 deletion was the most common, accounting for 7 out of 9 cases (**Table 3**).

**Table 2:** Genetic mutations identified in the whole cohort of patients developing malignancies and in the adult and pediatric sub-cohorts.

Genetic variants n (%)	Overall n= 52 (21%)	Adults n =24 (12.3%)	Pediatrics n = 28 (52.8%)
ADA	6	1	5
ATM	6	3	3
BTK	3	2	1
ELANE	3	0	3
MAGT1	3	2	1
CTLA4	2	2	0
PIK3CD	2	1	1
PIK3R1	2	0	2
WAS	3	1	2
CECR1	1	1	0
DCLRE1C	1	0	1
IFNGR1	1	0	1
FOXP3	1	1	0
IKZF1	1	0	1
NBN	1	0	1
NFKB1	1	1	0
NFKB2	1	1	0
RMRP	1	0	1
SH2D1A	4	0	4
TNFRSF13B het	6	5	1
TCF3 het	1	1	0
LIG1	1	1	0
FHL5 (STXBP2)	1	1	0
Unknown (n,%)	196 (79%)	171 (87.7%)	25 (47.2%)

the percentage of identified genetic mutations was calculated relative to the overall cohort and sub-cohorts, respectively.

**Table 3:** Cancer-related germline and somatic cytogenetic alterations identified in the whole cohort of patients developing malignancies and in the adult and pediatric sub-cohorts.

Germline cytogenetic alterations	Overall N = 9	Adults N = 3	Pediatrics N = 6
22q11 deletion	7	1	6
del 16p11.2	1	1	0
SF3B1 gene, trisomy chr 8	1	1	0
Somatic cytogenetic alterations	Overall N = 8	Adults N = 3	Pediatrics N = 5
Balanced inverted insertion of chromosome region 8q11.23-8q24 from one chromosome 8 into the long arm of one chromosome 19 at 19q13.13,	1	0	1
del(6)(q21),	1	0	1
Monosomy 7	1	0	1
Monosomy 7; Traslocation t(4;21)	1	0	1
Traslocation IgH/myc t(8;14)(q24;q32)	1	1	0
Traslocation t(9;22)	1	0	1
SS18	1	1	0
JAK2 (V617F variant)	2	2	0

### 3.7 Therapeutic management of IEIs and oncological treatments

The most common intervention for underlying immunodeficiencies or associated manifestations in patients who developed malignancies was immunoglobulin replacement therapy (IgRT), administered to 86.6% of adults and 54.7% of pediatric patients (n 198, 80%). Additionally, 16% of the cohort received anti-infective prophylaxis. Four ADA-SCID pediatric patients underwent gene therapy for their underlying IEI. With regard to the management of malignancies, treatment regimens varied: 123 tumors were treated with chemotherapy alone, while 46 patients received multimodal regimens that included immunotherapy and/or biologic agents. Allogeneic hematopoietic stem cell transplantation (HSCT) was performed in 28 cases of hematological malignancies (specifically NHL and acute leukemia). Treatment with HSCT was significantly more common in the pediatric cohort than in the adult cohort (35.8% vs. 4.4%, respectively; *p < 000.1*), reflecting the different therapeutic approaches between the two age groups. Comprehensive therapeutic data are summarized in **Table 4**.

**Table 4:** IEI-associated therapy and oncological treatments in the entire cohort and by age group.

Treatments	Overall N = 248	Adults N = 195	Pediatrics N = 53
IgRT, n (%)	198 (79.8%)	169 (86.7%)	29 (54.7%)
Immunosuppressive drugs, n (%)	19 (7.7%)	12 (6.2%)	7 (13.2%)
Growth Factors, n (%)	9 (3.6%)	6 (3.1%)	3 (5.7%)
Anti-infective prophylaxis, n (%)	40 (16.1%)	33 (16.9%)	7 (13.2%)
Gene therapy, n (%)	4 (1.6%)	0 (0%)	4 (7.5%)
Chemotherapy, n (%)	123 (49.6%)	87 (44.6%)	36 (67.9%)
Immunotherapy/biological drugs, n (%)	46 (18.5%)	31 (15.9.%)	15 (28.3%)
Radiotherapy, n (%)	51(20.6%)	40 (20.5%)	11(20.8%)
Surgery, n (%)	178 (71.8%)	153 (78.5%)	25 (47.2%)
HSCT, n (%)	28 (11.3%)	9 (4.6%)	19 (35.8%)

### 3.8 Tumor distribution and characteristics

#### 3.8.1 Pediatric cohort

During the study period, 53 separate cancers were diagnosed in as many pediatric patients. The most common malignancies were acute leukemias (13/53, 24.5%), comprising 10 lymphoblastic and 3 myeloid leukemias; the latter occurred in patients with severe congenital neutropenia (SCN), two of whom carried the somatic cytogenetic abnormality monosomy 7. These were followed by NHL (12/53, 22.6%) and HL (7/53, 13.2%). Of note, both these types of tumors occurred in the 0–14 age group. Other unspecified lymphoproliferative disorders (4/53) were also detected. Taken together hematologic neoplasms accounted for 68% of all tumors of this cohort. Soft tissue sarcomas and papillary thyroid carcinomas were observed in 4 patients each (7.5%), while central nervous system (CNS) tumors accounted for 5.6% of malignancies, consisting of two astrocytoma and one pineal germinoma. In addition, exceptionally rare malignancies in childhood, such as ovarian cancer in 3 cases (5.6%) and 1 case each of breast cancer, melanoma and testicular cancer, were diagnosed.

The comparison with normative data highlights that pediatric patients with IEIs display a remarkably different oncological profile compared to the general pediatric population. Specifically, NHL and HL occurred more frequently (22.6% vs. 6% and 13.2% vs. 7%, respectively), while the rates of lymphoblastic leukemia and CNS tumors were significantly lower than the normative data (18.8% vs. 33% and 5.6% vs. 20%, respectively, **Figure 5a**).

**Figure 5:**
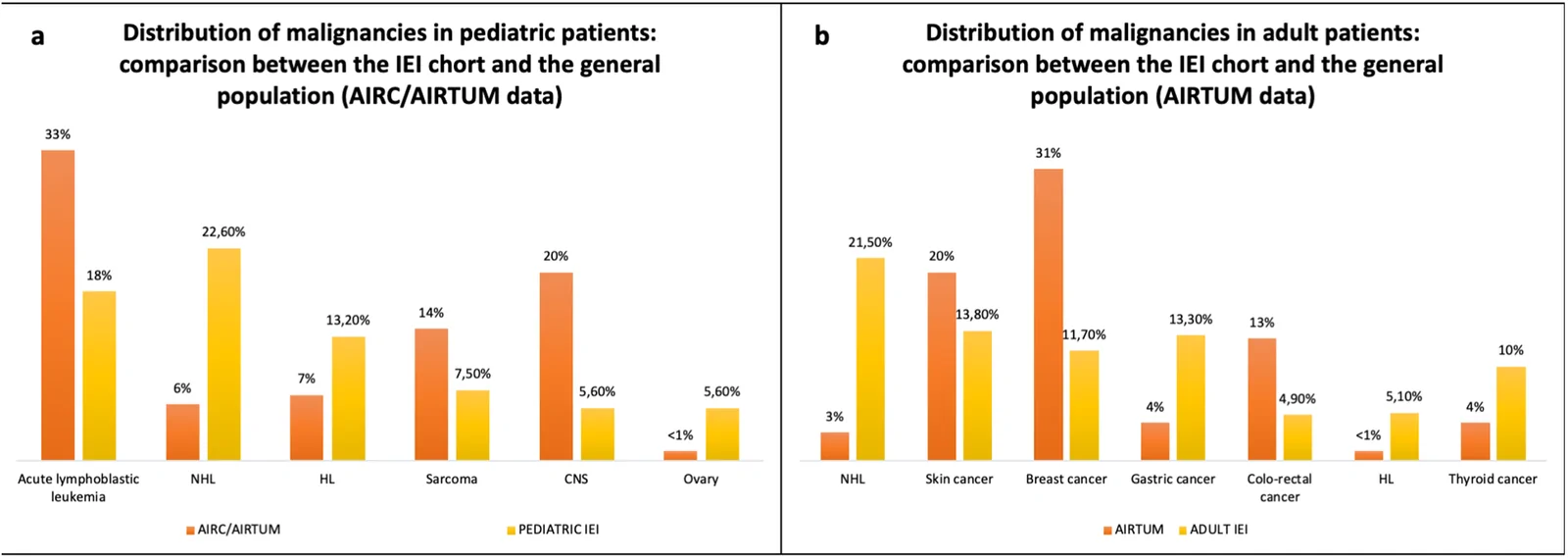
Distribution of malignancies: comparison between the IEI cohort and the general pediatric population **(a)** and adult general population **(b)** (AIRC/AIRTUM data). HL, Hodgkin’s lymphoma; NHL, non-Hodgkin’s lymphoma; CNS, Central Nervous System.

#### 3.8.2 Adult cohort

A total of 195 cancers were identified in the adult cohort. Contrary to Italian normative data (20, 21), which identify breast cancer as the most prevalent malignancy, our adult IEI cohort exhibited a distinct neoplastic distribution. Specifically, NHL (42 cases, 21.5%) and skin cancer (27 cases, 13.8%—comprising 10 cases of basal cell carcinoma, 9 of squamous cell carcinoma, and 8 of melanoma) emerged as the most frequently diagnosed tumors.

Furthermore, gastric adenocarcinoma (26 cases, 13.3%), breast cancer (23 cases, 11.7%), and thyroid cancer (20 cases, 10%) were frequently observed, followed by Hodgkin lymphoma and colorectal cancer, which accounted for 10 cases (5.1%). Collectively, solid tumors represented 70% of all malignancies within this cohort. A comprehensive comparison between IEI-associated adult cancers and AIRTUM data is presented in **Figure 5b**.

### 3.9 CVID cohort

CVID represented the largest subgroup among all IEIs enrolled in our study (1.398 patients out of 4.540, 30.8%). Within this sub-cohort, 163 patients (151 adults and 12 children) developed cancer, accounting for 65.7% (163/248) of all tumors.

The prevalence of malignancy was 11.7% (95% CI: 10.1 - 13.4), with an incidence rate of 7.3 cases per 1,000 person-years. The median age at cancer diagnosis was 49 years (range 4-77.9). Non-Hodgkin lymphoma (39 cases, 23.9%) and skin cancer (25 cases, 15.3%—including 10 cases of basal cell carcinoma, 9 of squamous cell carcinoma, and 6 of melanoma) emerged as the most frequently diagnosed tumors.

Furthermore, gastric cancer (24 cases, 14.7%), breast cancer (16 cases, 9.8%), and thyroid cancer (10 cases, 6.1%) were frequently observed, followed by HL and colorectal cancer, which accounted for 9 cases (5.5%) each. Of note, the median age at diagnosis of NHL and gastric cancer was 41 years (range: 6 - 81.9) and 51 years (range: 32-77.9) respectively, significantly lower than the expected in the general Italian population. Of interest, two cases of myeloproliferative disorders, polycythemia vera and essential thrombocythemia, both harboring the JAK2 V617F mutation, were also detected. Data on CVID cohort are summarized in **Table 5**.

**Table 5:** Demographic features and tumor distribution of the CVID cohort.

Features	CVID cohort 1.398 (30.8% of the whole cohort)
Patients with malignancies, n (%)	163 (65.7% of the malignancy cohort)
Sex, n (%)	F: 92 (56%) M: 71 (43%)
Adult, n (%)	151 (9%)
Children, n (%)	12 (7%)
Median Age (y) at onset of malignancy, (min, max)	49 (range 4-77.9)
Incidence of malignancy	7.3 cases per 1,000 person-years
Prevalence of malignancy	11.7% (95% CI: 10.1 - 13.4),
Patients who developed malignancy before IEI diagnosis, n (%)	44 (27%)
Malignancies, n (%)	Non-Hodgkin lymphoma: 39 (23.9)
	Skin cancer: 25 (15.3)
	Gastric cancer: 24 (14.7)
	Breast cancer: 16 (9.8)
	Thyroid cancer: 10 (6.1)
	Hodgkin lymphoma: 9 (5.5)
	Colorectal cancer: 9 (5.5)
	Acute lymphoblastic leukemia: 6 (3.7%)
	Bladder cancer: 4 (2.5%)
	Ovarian cancer: 3 (1.8%)
	Prostate cancer: 3 (1.8%)
	Uterine cancer: 3 (1.8%)
	CNS malignancies: 2 (1.2%)
	Lung cancer: 2 (1.2%)
	Myeloproliferative disorders: 2 (1.2%)
	Sarcoma: 2 (1.2%)
	Hepatocarcinoma: 1 (0.6%)
	Multiple myeloma: 1 (0.6%)
	Renal cancer: 1 (0.6%)
	Testicular cancer: 1 (0.6%)
Outcome, n (%)	Alive: 157 (96.3%)
	Dead: 6 (3.7%)

### 3.10 Survival and mortality

Survival was assessed for 240 out of 248 patients, comprising 193 adults and 47 children, whose outcome was known at the end of the study. During the prospective phase, 13 deaths were recorded: 8 in the adult group and 5 in the pediatric group, accounting for 4.1% and 10.6% of their respective cohorts. The leading cause of death was NHL, occurring in 10 out of 13 cases (76.9%), followed by gastric adenocarcinoma (2 cases) and breast cancer (1 case). Notably, all pediatric patients died of NHL (2 with XLP1, and 1 each with IFNGR1 deficiency, APDS2, and UnPAD, respectively). In the adult cohort, 6 patients with CVID died from NHL (4 cases) or gastric adenocarcinoma (2 cases). Additionally, two patients with UnPAD died, one from breast cancer and one from NHL. The causes of death were uncontrolled cancer, infections or multiple organ failure. The median age at death for the entire cohort was 38 years (range: 7–76) with an overall 10-year survival rate of 94% (95% CI: 90.2–96.4%) (**Figure 6**), whereas the median age for pediatric and adult patients was 12 years (range: 7–26) and 59 years (range: 29–76), respectively. When analyzed by cancer subtype, the 10-year survival rate was not significantly different between patients with hematologic malignancies and solid tumors which stood at 92% (95% CI: 84.7–96%) and 94% (95% CI: 89.1–96.8%) respectively (p=0.95) (**Figure 7a**). Likewise, no significant difference in the 10-year survival rate was found between the adult and pediatric cohorts: 95% (95% CI: 90.9–97.3%) vs. 90% (95% CI: 78–95.9%), respectively. p = 0.066 (**Figure 7b**). Finally, when comparing the CVID patient group with all other IEI categories, the 10-year survival rate for all tumors showed no significant difference: 95%,95% CI: 90.5–97.5%) vs. 91%, 95% CI: 82.2–95.6%), respectively p =0.46, **Figure 7c**).

**Figure 6:**
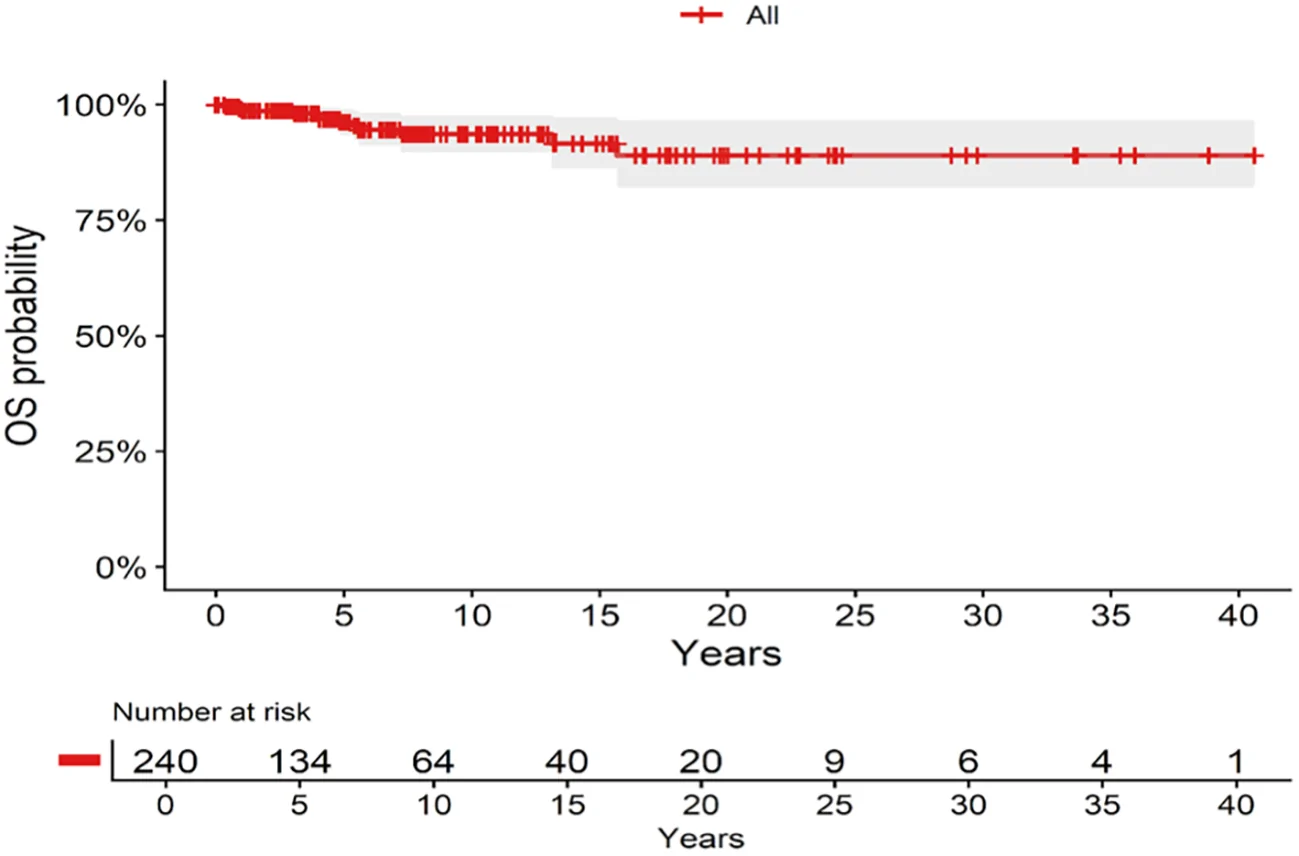
Overall cohort survival. The median age at death for the entire cohort was 38 years (range: 7–76) with an overall 10-year survival rate of 94% (95% CI: 90.2–96.4%).

**Figure 7:**
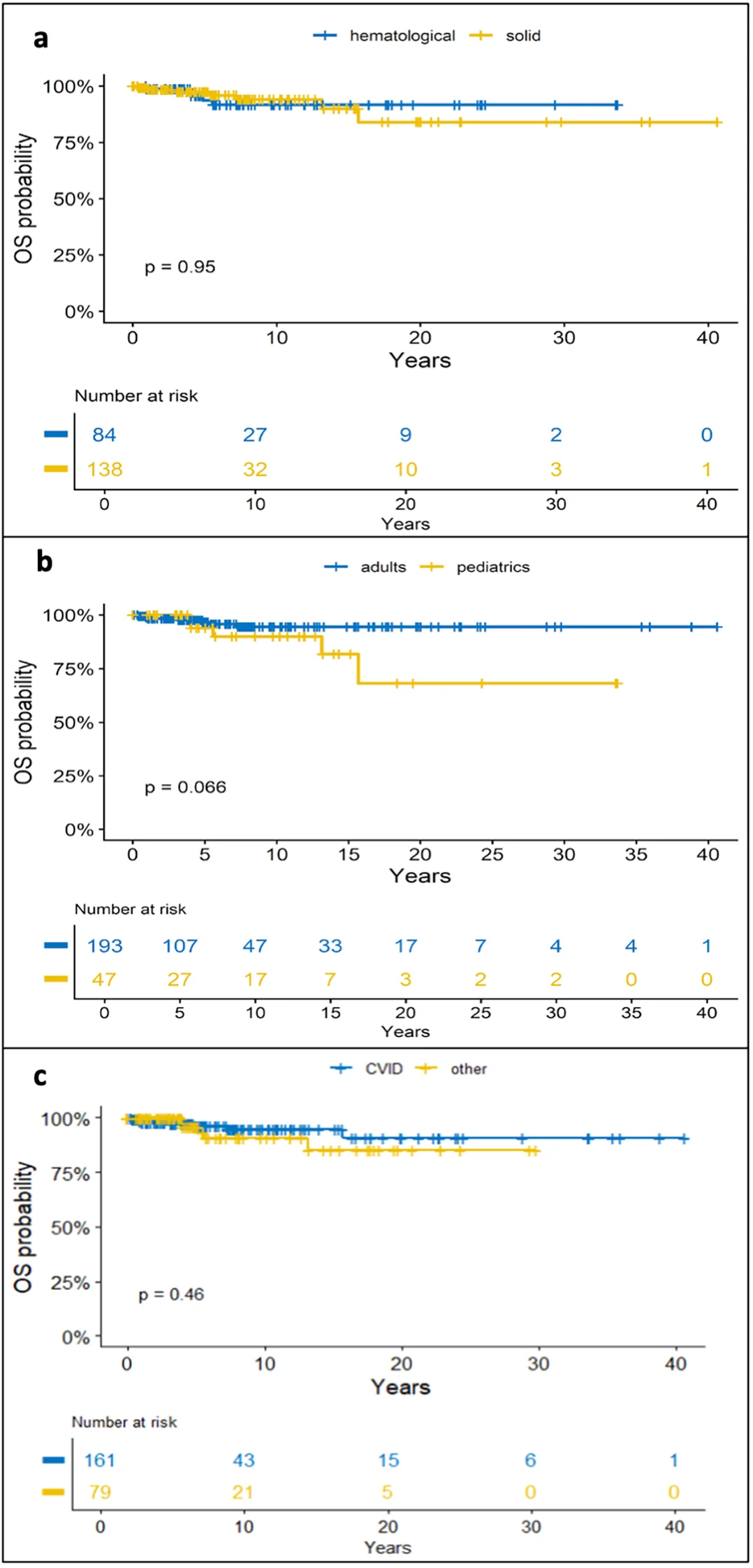
Comparison of the overall survival between patients with diagnosis of hematological versus solid malignancies **(a)**, adults and pediatric cohort **(b)**, and between CVID and all other IEI **(c)**. The 10-year survival rate was 92% (95% CI: 84.7–96.%) for those with hematologic cancers and 94% (95% CI: 89.1–96.8%) for patients with solid tumors (p=0.95)-**(a)**. No significant difference in the 10-year survival rate was observed between the adult and pediatric cohort 95% (95% CI: 90.9–97.3%) vs. 90% (95% CI: 78–95.9%), respectively. p = 0.066 **(b)**. The 10-year survival rate showed no significant differences between CVID and patients with other IEIs (95% ,95% CI: 90.5–97.5%) vs. 91%, 95% CI: 82.2–95.6%), respectively p =0.46 **(c)**.

## 4 Discussion

This retrospective-prospective multicenter study provides a comprehensive overview of the prevalence, clinical characteristics, and outcomes of malignancies in a large Italian cohort of patients with IEIs, offering a real-world representation of cancer burden across both adult and pediatric populations. Overall, 248 malignancies were identified in 248 out of 4540 IEI patients, the majority of which cases occurring patients with PAD, particularly CVID, reflecting both their numerical predominance and longer life expectancy compared to other IEI categories. Combined immunodeficiencies associated with syndromic features or DNA repair defects, such as 22q11.2 deletion syndrome and AT, as well as ADA-SCID, were also well represented and characterized by an early onset of malignancy. Notably, no cases of cancer were observed among patients with bone marrow failure syndromes, CGD, or complement deficiency during the study period, confirming the data showing that not all IEIs present an equal susceptibility to the development of cancer (22–24).

The overall cancer prevalence in our cohort was comparable to that reported in the general Italian population and in the European region but significantly lower than estimates from the ESID registry (14, 15, 20). This apparent discrepancy may be explained by differences in cohort composition, in terms of age groups and IEI categories or survivorship bias. Importantly, despite similar prevalence rates, the age at cancer onset in our cohort was consistently younger than in the general population, particularly in patients with AT and Del22 syndrome, supporting the notion of an intrinsic cancer predisposition driven by altered immune checkpoint regulation and genetic instability (17, 23).

Consistent with previous reports, nearly one-third of patients presented with malignancy as the first clinical manifestation of IEI (3, 14, 16). In this subgroup, the underlying IEIs were predominantly represented by CVID, AT, and specific combined immunodeficiencies, with immune dysregulation or recurrent infections being recognized only retrospectively. This finding underscores the importance of considering an underlying immunodeficiency in patients presenting with early-onset, atypical, or recurrent malignancies, and highlights the need for increased awareness among oncologists and other specialists (11, 25).

As in the general population, our data indicate distinct oncological profiles between the pediatric and adult cohorts, with a marked prevalence of hematologic and solid tumors, respectively, in the two groups. However, the distribution of tumor types differed significantly from that observed in the general population (20, 21). Hematologic malignancies predominated in the IEI cohort, particularly lymphoid neoplasms, which accounted for the majority of cases. NHL, HL, and acute leukemia, taken together, were the most frequent diagnoses, consistent with data from international registries and recent meta-analyses (16, 26). These findings likely reflect the central role of immune dysregulation, impaired lymphocyte development, chronic antigenic stimulation, and defective immunosurveillance in lymphomagenesis (27). With respect to solid tumors, skin (primarily non melanoma), gastric, thyroid, and breast cancers were the most common. This distribution contrasts with Italian normative data where breast, lung and colorectal cancers are more prevalent (20, 21), and these differences likely reflect a distinct molecular pattern implicated in oncogenesis in IEI patients.

Consistently with the molecular heterogeneity of IEIs and cancer pathogenesis, the type of tumors, the prevalence estimations, and recorded ages at malignancy diagnosis varied significantly depending on the specific IEI subtype. CVID was the largest subgroup and, consistent with previous studies (28, 29), exhibited the highest cancer prevalence, which was approximately double that of the overall cohort. In these patients, malignancies, primarily represented by NHL and skin cancer, occurred at a later age compared to other IEIs but still earlier than in the general population. While other IEI categories in our study are frequently linked to specific monogenic defects, CVID predominantly represents a condition of complex immune dysregulation.

The increased cancer risk in this cohort appears to be less driven by a single genetic mutation than by the synergistic effects of profound immunodeficiency against tumor cells, chronic mucosal inflammation, and persistent infections—especially *Helicobacter pylori*—thereby supporting a role for impaired mucosal immunity in carcinogenesis (28, 29).

Pediatric IEI patients displayed a markedly distinct oncologic profile compared to the general population (21). In our cohort, lymphomas and acute leukemias were significantly overrepresented, whereas neuroblastoma, which is the most frequent cancer in the 0–5 age group according to the AIRC report, was absent. This absence aligns with the embryogenetic origin of neuroblastoma from neural crest cells, a lineage whose oncogenesis is typically independent of the immune dysregulation or genomic instability pathways characteristic of IEIs. The occurrence of typically rare tumors in childhood, including thyroid and ovarian cancers, further supports the presence of early genetic susceptibility and highlights IEI as an important predisposing condition for pediatric malignancies. Additionally, the development of myeloid malignancies in patients with severe congenital neutropenia confirms the well-established association between specific cytogenetic abnormalities, such as monosomy 7 and clonal evolution (30).

Moreover, the high prevalence of comorbidities—particularly autoimmune diseases, chronic lung and gastrointestinal disorders and viral infections, mostly EBV related, supports the role of chronic immune activation and persistent inflammatory stimuli as cofactors in tumorigenesis (31, 32).

Although genetic testing was not available for all patients, pathogenic variants were identified in a substantial proportion of those tested suggesting that the underlying molecular defect may contribute, in these cases, directly to both immunodeficiency and cancer susceptibility.

Despite the increased cancer risk, the overall prognosis in our cohort was relatively favorable, with a 10-year survival rate of 94% at the end of the study time. Survival did not significantly differ between pediatric and adult patients, nor between solid and hematologic malignancies or between CVID and all other IEI patients. Even when considering specific high-risk subgroups, such as patients with AT, no statistically significant difference in survival was observed, likely due to the small sample size of this sub-cohort. These findings may reflect improvements in both oncological treatments and supportive care in IEI patients. However, they contrast with some previous reports, such as data from the Iranian registry, which described worse outcomes for hematologic malignancies (22). Differences in cohort composition, access to care, and treatment strategies may account for these discrepancies.

The management of malignancies in IEI remains challenging, requiring a careful balance between treatment efficacy and toxicity, alongside consideration of the underlying immune defect. Patients face an elevated risk of suboptimal treatment response, therapy-related complications and relapse (11) Despite this, standardized guidelines are currently limited to specific IEI subtypes, particularly those involving DNA repair defects (33, 34). In Italy, surveillance strategies such as annual upper endoscopy and lymph node ultrasound in CVID patients have been proposed, but broader, evidence-based guidelines are still lacking. Our findings highlight the critical need for tailored screening and management protocols, alongside a multidisciplinary approach, to optimize long-term clinical outcomes and mitigate treatment-related toxicities in this vulnerable patient population.

## 5 Limitations and strengths of the study

This study has some limitations. The partially retrospective design of this study may have introduced selection and information bias. Furthermore, genetic data were not available for all patients, thereby limiting genotype–phenotype correlation analysis. In addition, bone marrow failure syndromes are likely underrepresented, as these patients are often managed in hematology–oncology settings, leading to potential referral bias. However, the study also has notable strengths. The multicenter, registry-based design allowed inclusion of a large and heterogeneous cohort of adult and pediatric IEI patients, reflecting real-world clinical practice. Integration with national registries enabled a reliable estimation of the number of IEI patients and a direct comparison with cancer data from the general population, while the longitudinal dataset provided valuable insights into the cancer spectrum and outcomes across IEI subtypes.

## 6 Conclusion

In this large Italian IEI cohort, the overall cancer prevalence was comparable to that of the general population, however the risk varied across IEI subtypes, with distinct tumor patterns and earlier onset. Malignancies were primarily represented by non -Hodgkin lymphoma and skin cancers. CVID patients showed the highest cancer prevalence exceeding both other IEI subgroups and the general population, which underscores the need for tailored surveillance strategies. Early diagnosis of IEI is crucial for timely interventions, including immunomodulatory therapies, hematopoietic stem cell transplantation, or gene therapy. Integrating genetic testing, infection monitoring, and personalized, risk-based cancer screening into routine care, combined with a multidisciplinary approach, is essential for optimal patient management and the best clinical outcomes.

## Data Availability

The original contributions presented in the study are included in the article/supplementary material, further inquiries can be directed to the corresponding author/s.

## References

[1] Poli MC Aksentijevich I Bousfiha AA Cunningham-Rundles C Hambleton S Klein C . Human inborn errors of immunity: 2024 update on the classification from the International Union of Immunological Societies Expert Committee. J Hum Immun. (2025) 1:e20250003. doi: 10.70962/jhi.20250003 41608114 PMC12829761

[2] Bousfiha AA Jeddane L Moundir A Poli MC Aksentijevich I Cunningham-Rundles C . The 2024 update of IUIS phenotypic classification of human inborn errors of immunity. J Hum Immun. (2025) 1:e20250002. doi: 10.70962/jhi.20250002 41608113 PMC12829316

[3] Hauck F Voss R Urban C Seidel MG . Intrinsic and extrinsic causes of Malignancies in patients with primary immunodeficiency disorders. J Allergy Clin Immunol. (2018) 41:59–68.e4. doi: 10.1016/j.jaci.2017.06.009 28669558

[4] Bosch JW Hlaváčková E Derpoorter C Fischer U Saettini F Ghosh S . How to recognize inborn errors of immunity in a child presenting with a Malignancy: guidelines for the pediatric hemato-oncologist. Pediatr Hematol Oncol. (2023) 40:131–46. doi: 10.1080/08880018.2022.2085830 35913104

[5] Bakhuizen JJ van Dijk F Bon SBB van der Veken LT Huibers MMH van Montfrans JM . Exploring germline variants in genes associated with inborn errors of immunity and inherited bone marrow failure syndromes in pediatric hematological Malignancies. Eur J Cancer. (2025) 226:115598. doi: 10.1016/j.ejca.2025.115598 40633199

[6] Kindler O Quehenberger F Benesch M Seidel MG . The Iceberg Map of germline mutations in childhood cancer: focus on primary immunodeficiencies. Curr Opin Pediatr. (2018) 30:855–63. doi: 10.1097/MOP.0000000000000680 30124581

[7] Abolhassani H Wang Y Hammarström L Pan-Hammarström Q . Hallmarks of cancers: Primary antibody deficiency versus other inborn errors of immunity. Front Immunol. (2021) 17:720025. doi: 10.3389/fimmu.2021.720025 34484227 PMC8416062

[8] Cohen JI . Primary immunodeficiencies associated with EBV disease. Curr Top Microbiol Immunol. (2015) 390:241–65. doi: 10.1007/978-3-319-22822-8_10 26424649 PMC6349415

[9] Fournier B Latour S . Immunity to EBV as revealed by immunodeficiencies. Curr Opin Immunol. (2021) 72:107–15. doi: 10.1007/82_2025_316 33989894

[10] Dropulic LK Cohen JI . Severe viral infections and primary immunodeficiencies. Clin Infect Dis. (2011) 53:897–909. doi: 10.1093/cid/cir610 21960712 PMC3189169

[11] Roberti G Maestrini G Polito B Amato L Parolo E Casazza G . Inborn errors of immunity in pediatric hematology and oncology: Diagnostic principles for clinical practice. J Clin Med. (2025) 14:6295. doi: 10.3390/jcm14176295 40944054 PMC12429287

[12] Kiykim A Eker N Surekli O Nain E Kasap N Aktürk H . Malignancy and lymphoid proliferation in primary immune deficiencies; hard to define, hard to treat. Pediatr Blood Cancer. (2020) 67:e28091. doi: 10.1002/pbc.28091 31736244

[13] Moratti M Rivalta B Cardoni A Santilli V Attardi E Manno EC . Lymphoproliferation in inborn errors of immunity: From challenging diagnosis to histologic revision. J Hum Immun. (2026) 2:e20250174. doi: 10.70962/jhi.20250174 42170321 PMC13177383

[14] Bogaert DJA Wolfsberger CH Attarbaschi A Gathmann J Warnatz K Mueller G . Epidemiology and management of Malignancies in patients with inborn errors of immunity: an ESID registry study of 19,959 patients. J Allergy Clin Immunol. (2026) 157:739–53. doi: 10.1016/j.jaci.2025.10.033 41242640

[15] De Angelis R Demuru E Baili P Troussard X Katalinic A Chirlaque Lopez MD . Complete cancer prevalence in Europe in 2020 by disease duration and country (EUROCARE-6): a population-based study. Lancet Oncol. (2024) 25:293–307. doi: 10.1016/S1470-2045(23)00646-0 38307102

[16] Fekrvand S Abolhassani H Esfahani ZH Fard NNG Amiri M Salehi H . Cancer trends in inborn errors of immunity: A systematic review and meta-analysis. J ClinImmunol. (2024) 45:34. doi: 10.1007/s10875-024-01810-w 39466473

[17] Bomken S van der Werff Ten Bosch J Attarbaschi A Bacon CM Borkhardt A Boztug K . Current understanding and future research priorities in Malignancy associated with inborn errors of immunity and DNA repair disorders: The perspective of an interdisciplinary working group. Front Immunol. (2018) 12:2912. doi: 10.3389/fimmu.2018.02912 30619276 PMC6299915

[18] Pastorczak A Attarbaschi A Bomken S Borkhardt A van der Werff ten Bosch J Elitzur S . Consensus recommendations for the clinical management of hematological Malignancies in patients with DNA double stranded break disorders. Cancers (Basel). (2022) 14:2000. doi: 10.3390/cancers14082000 35454905 PMC9029535

[19] Seidel MG Kindle G Gathmann B Quinti I Buckland M van Montfrans J . The European society for immunodeficiencies (ESID) registry working definitions for the clinical diagnosis of inborn errors of immunity. J Allergy Clin Immunol Pract. (2019) 7:1763–70. doi: 10.1016/j.jaip.2019.02.004 30776527

[20] AIOM AIRTUM Fondazione AIOM ONS PASSI PASSI d’Argento . I numeri del cancro in talia 2024. Roma: AIOM (2024). Available online at: https://www.registri-tumori.it (Accessed August 07, 2026).

[21] Associazione Italiana per la Ricerca sul Cancro (AIRC) . Le statistiche del cancro in Italia. Available online at: https://www.airc.it/cancro/informazioni/cosa-e-il-cancro (Accessed August 07, 2026).

[22] Delavari S Wang Y Moeini Shad T Pashangzadeh S Nazari F Salami F . Clinical and immunologic characteristics of non-hematologic cancers in patients with inborn errors of immunity. Cancers (Basel). (2023) 15:764. doi: 10.3390/cancers15030764 36765721 PMC9913767

[23] Nakano Y Kuiper RP Nichols KE Porter CC Lesmana H Meade J . Update on recommendations for cancer screening and surveillance in children with genomic instability disorders. Clin Cancer Res. (2024) 30:5009–20. doi: 10.1158/1078-0432.CCR-24-1098 39264246 PMC11705613

[24] Tavakol M Delavari S Salami F Ansari S Rasouli SE Chavoshzadeh Z . Diversity of Malignancies in patients with different types of inborn errors of immunity. Allergy Asthma Clin Immunol. (2022) 18:106. doi: 10.1186/s13223-022-00747-2 36510326 PMC9743521

[25] Kindle G Alligon M Albert MH Buckland M Edgar JD Gathmann B . Inborn errors of immunity: Manifestation, treatment, and outcome-an ESID registry 1994-2024 report on 30,628 patients. J Hum Immun. (2025) 1:e20250007. doi: 10.70962/jhi.20250007 41347188 PMC12674179

[26] Mayor PC Eng KH Singel KL Abrams SI Odunsi K Moysich KB . Cancer in primary immunodeficiency diseases: Cancer incidence in the United States Immune Deficiency Network Registry. J Allergy Clin Immunol. (2018) 141:1028–35. doi: 10.1016/j.jaci.2017.05.024 28606585 PMC5723251

[27] Sharma S Pilania RK Anjani G Sudhakar M Arora K Tyagi R . Lymphoproliferation in inborn errors of immunity: The eye does not see what the mind does not know. Front Immunol. (2022) 4:856601. doi: 10.3389/fimmu.2022.856601 35603189 PMC9114776

[28] Pedini V Verga JU Terrenato I Menghini D Mezzanotte C Danieli MG . Incidence of Malignancy in patients with common variable immunodeficiency according to therapeutic delay: an Italian retrospective monocentric cohort study. Allergy Asthma Clin Immunol. (2020) 16:54. doi: 10.1186/s13223-020-00451-z 32944022 PMC7491341

[29] Pulvirenti F Pecoraro A Cinetto F Milito C Valente M Santangeli E . Gastric cancer is the leading cause of death in Italian adult patients with common variable immunodeficiency. Front Immunol. (2018) 9:2546. doi: 10.3389/fimmu.2018.02546 30455695 PMC6230622

[30] Pezeshki A Podder S Kamel R Corey SJ . Monosomy 7/del (7q) in inherited bone marrow failure syndromes: A systematic review. Pediatr Blood Cancer. (2017) 64:10. doi: 10.1002/pbc.26714 28708320 PMC5937691

[31] Tiri A Masetti R Conti F Tignanelli A Turrini E Bertolini P . Inborn errors of immunity and cancer. Biol (Basel). (2021) 10:313. doi: 10.3390/biology10040313 33918597 PMC8069273

[32] Segura-Tudela A Nieto-López C Bermejo-Olivera FJ Andara LA Arroyo-Ródenas J Dueñas-Prieto L . Malignancy and autoimmune susceptibility in adult patients with human inborn errors of immunity. J ClinImmunol. (2025) 45:136. doi: 10.1007/s10875-025-01935-6 41026241 PMC12484250

[33] Bröckelmann PJ de Jong MRW Jachimowicz RD . Targeting DNA repair, cell cycle, and tumor microenvironment in B cell lymphoma. Cells. (2020) 9:2287. doi: 10.3390/cells9102287 33066395 PMC7602196

[34] Tabori U Hansford JR Achatz MI Kratz CP Plon SE Frebourg T . Clinical management and tumor surveillance recommendations of inherited mismatch repair deficiency in childhood. Clin Cancer Res. (2017) 23:e32–7. doi: 10.1158/1078-0432.CCR-17-0574 28572265

